# Comprehensive Evaluation of Deep Coal Miners’ Unsafe Behavior Based on HFACS-CM-SEM-SD

**DOI:** 10.3390/ijerph191710762

**Published:** 2022-08-29

**Authors:** Li Yang, Xue Wang, Junqi Zhu, Liyan Sun, Zhiyuan Qin

**Affiliations:** School of Economics and Management, Anhui University of Science and Technology, Huainan 232001, China

**Keywords:** deep coal mine, unsafe behavior, sensitivity analysis, SEM-SD, comprehensive evaluation

## Abstract

The unsafe behavior of miners seriously affects the safety of deep mining. A comprehensive evaluation of miners’ unsafe behavior in deep coal mines can prevent coal mine accidents. This study combines HFACS-CM, SEM, and SD models to evaluate miners’ unsafe behaviors in deep coal mining. First, the HFACS-CM model identifies the risk factors affecting miners’ unsafe behavior in deep coal mines. Second, SEM was used to analyze the interaction between risk factors and miners’ unsafe behavior. Finally, the SD model was used to simulate the sensitivity of each risk factor to miners’ unsafe behavior to explore the best prevention and control strategies for unsafe behavior. The results showed that (1) environmental factors, organizational influence, unsafe supervision, and unsafe state of miners are the four main risk factors affecting the unsafe behavior of miners in deep coal mines. Among them, the unsafe state of miners is the most critical risk factor. (2) Environmental factors, organizational influence, unsafe supervision, and the unsafe state of miners have both direct and indirect impacts on unsafe behaviors, and their immediate effects are far more significant than their indirect influence. (3) Environmental factors, organizational influence, and unsafe supervision positively impact miners’ unsafe behavior through the mediating effect of miners’ unsafe states. (4) Mental state, physiological state, business abilities, resource management, and organizational climate were the top five risk factors affecting miners’ unsafe behaviors. Taking measures to improve the adverse environmental factors, strengthening the organization’s supervision and management, and improving the unsafe state of miners can effectively reduce the risk of miners’ unsafe behavior in deep coal mines. This study provides a new idea and method for preventing and controlling the unsafe behavior of miners in deep coal mines.

## 1. Introduction

Coal plays a vital role across industries as the most critical global energy source. However, the frequent occurrence of coal mine accidents poses significant challenges to the safe production of coal mining enterprises. Heinrich’s theory of accident causes posits that accidents are always closely related to the unsafe behaviors of people involved and the unsafe state of objects in the environment [1]. With the development of science and technology, the unsafe conditions causing accidents have gradually improved, but the unsafe behaviors of people that can cause accidents still exist. Relevant studies also point out that in a variety of coal mine accidents in China, the majority of coal mine accidents are caused by workers’ unsafe behavior [2]. As the largest coal producer and consumer globally, China has gradually entered the stage of deep coal mining in the central and eastern regions, as shallow coal resources have been exhausted in recent years. The extremely high temperature and humid working environment in deep mines significantly affects the safety state of miners, causing them to exhibit various unsafe behaviors and leading to accidents [3]. In recent years, accidents in deep coal mines have occurred frequently in China, causing heavy casualties and severe property losses. For example, in June 2020, an accident occurred at a mining depth of 950 m in a newly built coal mine in Tokkuzliu County, China, resulting in one death and a direct economic loss of 1.34 million yuan. In October 2021, a coal warehouse collapse occurred at Yinxing Coal Industry Co., Ltd. of the Ningxia Coal Industry in China at a mining depth of 1110 m, resulting in one death and a direct economic loss of 1.7052 million yuan. In addition, scholars have pointed out that unsafe behaviors account for more than 90% of the risk caused by gas outbursts, gas explosions, and mine water accidents [4,5]. Therefore, it is essential to comprehensively evaluate miners’ unsafe behavior in deep coal mines and explore effective measures to reduce the risk of miners’ unsafe behavior.

Scholars have conducted many studies evaluating coal miners’ unsafe behavior. The HFACS-CM (Human Factor Analysis and Classification System for Coal Mines) is the most common research method for analyzing miners’ unsafe behaviors in coal mine accidents, which can identify the risk factors affecting miners’ unsafe behavior at different levels [6,7]. Liu used the HFACS-CM to classify the factors affecting miners’ unsafe behaviors and formulated a prevention and control system for the miners’ unsafe behaviors [8]. In addition, some scholars have used the improved HFACS-CM framework and intelligent algorithm to point out the relationship between risk factors and miners’ unsafe behavior, revealing four causative routes leading to coal mine safety accidents [9]. The SEM (Structural Equation Model), SD (Systems Dynamics) model, and BLR (Binary Logistics Regression) model have also been used to evaluate miners’ unsafe behaviors [10,11,12]. Cheng et al. used SEM to study the influence of leadership behavior on miners’ work safety behavior [13]. Yu et al. used the SD model to simulate miners’ unsafe behavior intervention strategies based on individual factors, physical environment factors, safety leadership, and other factors to explore the best intervention strategy [14]. Wang et al. introduced the BLR method to analyze the relationship between different influencing factors and miners’ unsafe behaviors [12]. Compared to shallow mines, the risk factors affecting miners’ unsafe behaviors in deep coal mines are more complex and diverse [15]. The main characteristics of a deep coal mining environment are high temperatures and high humidity [16,17]. The body’s core temperature is maintained at about 37 °C, above which the body exhibits various adverse reactions [18]. Through comparative experiments, E.K. et al. also pointed out that in industrial environments with 24 °C WBGT above and core temperature above 38 °C, workers’ unsafe behaviors and accidents increased with work efficiency [19]. Scholars have pointed out that the central nervous system of workers working in the sultry, humid environment, and slow ventilation in the deep coal mine is unstable [20]. Roy et al. also pointed out that with the increase in coal mining depth, the increased thermal stress in deep mines seriously threatens miners’ health, production, and safety [21]. In addition, relevant studies have confirmed that the hot and humid underground climate in deep mines not only seriously affects the physical and mental health of miners but also reduces the production efficiency of miners and increases the safety risk of deep mining [22]. Li et al. also showed through a mouse biological simulation model experiment (CEBS) that environmental factors such as temperature, humidity, and noise in coal mine workplaces could lead to adverse reactions, such as anxiety, depression, and impaired memory ability in mice [23]. From the above analysis, the harsh working environment, such as high temperature, high humidity, noise, and dust in the underground coal mine, combined with high-intensity physical labor, will cause coal miners to suffer from various occupational diseases and destructive emotions. These unhealthy physical and mental states are more likely to lead to unsafe behaviors among coal miners [14,24,25]. Therefore, it is challenging to evaluate miners’ unsafe behaviors in deep coal mines using traditional evaluation methods [26,27].

Although scholars have adopted various research methods to evaluate the unsafe behaviors of coal miners, the objects of these studies are all the unsafe behaviors of miners in shallow coal mines. There are few studies on the unsafe behaviors of miners in deep coal mines. As a result, the influencing mechanism of various risk factors on miners’ unsafe behavior in deep coal mines is still unclear. SEM, SD, and game theory are commonly used to analyze the relationship between risk factors and miners’ unsafe behavior in shallow coal mines [10,11,28]. These methods have their disadvantages and can only be used under certain conditions. In addition, the effect of various risk factors on miners’ unsafe behavior in deep coal mines is complicated. Therefore, using these methods alone makes it difficult to accurately describe the action mechanism of each risk factor in miners’ unsafe behavior in deep coal mines. Based on the HFACS-CM, SEM, and SD models, this study constructs the HFACS-CM-SEM-SD model to evaluate miners’ unsafe behavior in deep coal mines. First, based on the HFACS-CM model framework, the risk factors affecting miners’ unsafe behavior in deep coal miners are identified. Second, the SEM model was used to analyze the interaction between risk factors and miners’ unsafe behavior. Finally, the SD model is used to simulate a strategy to reduce the risk of miners’ unsafe behavior. This study will fill the gap in the current field, reveal the influence mechanism of various risk factors on miners’ unsafe behaviors, and provide new ideas for preventing, controlling, and intervening in miners’ unsafe behaviors in deep coal mines.

## 2. Materials and Methods

### 2.1. Research Framework

**Figure 1:**
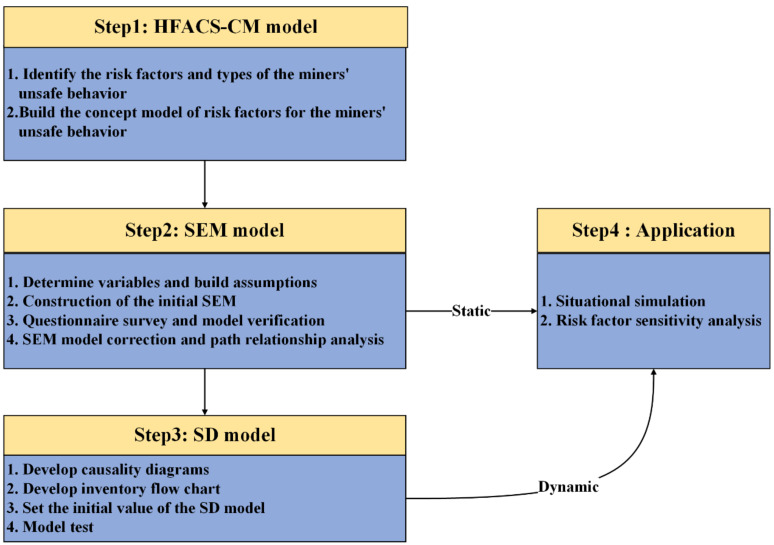
Research framework and process of this study.

In 1956, United States Professor J. W. Forrester of the United States proposed SD (System Dynamics) to analyze production management, inventory management, and other enterprise management issues [29]. This mathematical modeling technique is used to understand and discuss complex problems and their changes over time. It has been widely applied in many fields, such as maritime affairs, engineering construction, education, and behavioral science [30,31,32,33]. SD has outstanding advantages in analyzing dynamic complex systems with internal feedback loops, and its application in miners’ unsafe behavior has been well developed and quite mature. This model can provide a way to analyze the dynamic relationship between the safety input and unsafe behavior levels of deep coal mines [34,35]. At the same time, the SD model is based on a feedback loop to study the problem, and the data dependence is not high. Most parameters in the model have little influence on the simulation system. The simulation system can run stably as long as there are loops in the model, and the relationship among the factors is reasonable. Therefore, using the SD model to evaluate miners’ unsafe behavior in the deep coal miner can overcome the problems of risk uncertainty of miners’ unsafe behavior and insufficient data on risk factors. However, it is challenging to identify the variables of the SD model and quantify the equations between the variables. To solve this problem, we introduced the HFACS-CM model, which can identify the risk factors of miners’ unsafe behavior in a deep coal mine, and SEM, which can explain the relationship between variables. The analysis results of these two models support the establishment of the SD model. Compared with the traditional multiple regression model, the combination of HFACS-CM and SEM can quickly identify the risk factors of unsafe behaviors of miners, test the internal structural relations between variables in the model, and visualize these relationships [8,36]. Although both HFACS-CM and SEM are static research methods, SD models can be established according to the correlation between the risk factors identified by HFACS-CM and the factors analyzed by SEM. Therefore, the SD model based on HFACS-CM and SEM was found to quantitatively predict the development trend of the unsafe behavior level of deep coal miners and the sensitivity of each risk factor to the unsafe behavior level of coal miners. As shown in Figure 1, this study was divided into four steps.

The first step was the HFACS-CM modeling process. Based on expert interviews, deep coal mine accident analysis, and a literature review, the HFACS-CM model was used to identify the risk factors for the unsafe behavior of deep coal miners. Subsequently, the main types of unsafe behavior of miners were determined. Finally, a conceptual model of risk factors for the unsafe behavior of deep coal miners was constructed.

The second step was the SEM modeling process. This step mainly included determining latent and observation variables, a questionnaire survey, data validation, SEM model construction, and path coefficient output. First, according to the conceptual model of risk factors, the risk factors affecting the unsafe behavior of deep coal miners are determined. The survey scale and questionnaires were then compiled. Second, based on the survey data from the questionnaire, the interaction between risk factors affecting the unsafe behavior of miners was analyzed. Third, according to the data analysis results, the unsafe behavior SEM of deep coal mine workers was constructed. Finally, the path coefficients of each factor were obtained by evaluating the goodness-of-fit of the initial SEM model.

The third step was the SD modeling process. Based on the interaction between variables, a causal cycle diagram, which helps to understand deep coal mines’ safety input and the level of unsafe behaviors of miners, was first established. Second, Vensim simulation software was used to transform the causal cycle diagram into an inventory flow chart, and the initial parameters and equations were assigned to the corresponding variables. Finally, the adequacy of the relationships between the variables was verified through model validation.

The fourth step was to apply the HFACS-CM-SEM-SD model. In the dynamic simulation process, different scenarios were simulated to measure the influence of varying coal mine safety inputs on the level of unsafe behavior of miners.

### 2.2. HFACS-CM Model

Based on the Swiss cheese model, Shappell and Wiegmann established the HFACS model, which can identify vulnerabilities in cheese [37]. It has been widely applied in various fields, such as construction projects, air traffic accidents, the medical industry, and the mining industry [6,38,39,40]. Liu et al. used AHP combined with HFACS-CM to analyze the unsafe behavior of miners in significant coal mine accidents [6]. Liu et al. analyzed the influencing factors of miners’ unsafe behaviors using the HFACS and SEM models and pointed out the importance of the factors. [8]. Fa et al. combined the HFACS model and intelligent algorithm to study the unsafe behavior of miners at both the individual and organizational levels [9]. Although the factors in the original HFACS-CM model are useful, some are not entirely applicable to deep coal mines, and the model should be improved according to the specific conditions of deep coal mines and the characteristics of miners [41,42]. This study aimed to identify the risk factors that specifically influence the unsafe behavior of deep coal miners. Therefore, this study modified unsafe behavior in the original HFACS-CM model. The unsafe behavior in the modified model mainly refers to the unsafe behavior of the front-line workers in the deep coal mine and the unsafe behavior of the related managers. In the improved HFACS-CM model, problems at higher levels may lead to problems at lower levels and workers’ unsafe behavior. This model is more suitable for comprehensively identifying the risk factors for the unsafe behaviors of miners in deep coal miners.

**Figure 2:**
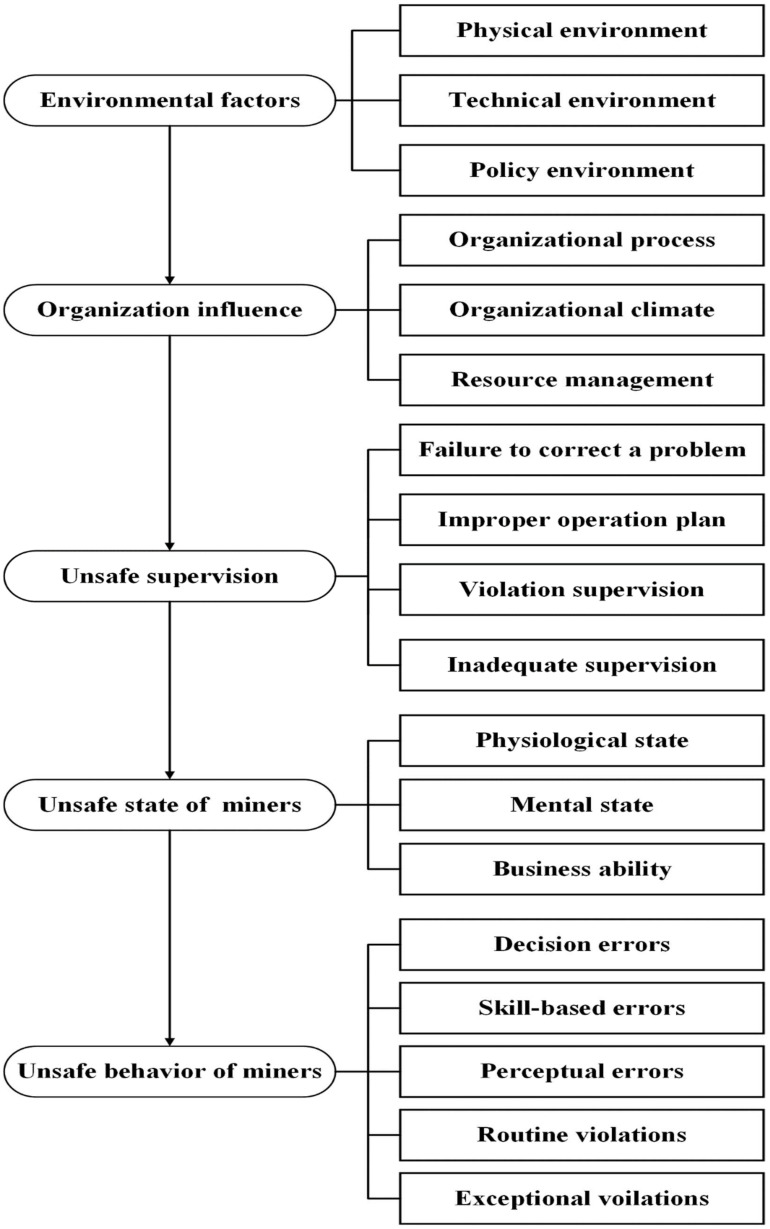
Improved HFACS-CM model of unsafe behavior of deep coal miners.

In deep coal mining, geological structure, extremely high temperature, substantial humidity, and other environmental factors affect the physiology and psychology of workers, leading to unsafe behavior [14,43]. In addition, with the continuous progress of mining technology and the rapid development of society, the behavior of coal miners is also increasingly affected by the external environment, such as information technology, the economy, politics, etc. Therefore, in this study, the environmental factors in the preconditions of unsafe behaviors in the original HFACS model were moved to the first layer. This new first layer consisted of physical, technical, and policy environments, which were summarized as environmental factors. The premise of unsafe behavior in the original HFACS model is changed to the unsafe state of miners, including workers’ psychological and physiological states and business abilities. Realizing coal mine production goals requires cooperation and communication between team members. Therefore, this study added teamwork and communication based on the original HFACS model and classified them into the organizational climate category. The improved HFACS-CM model is shown in Figure 2.

As shown in Figure 2, the improved HFACS-CM model divided the risk factors into five levels: environmental factors, organizational influence, unsafe supervision, unsafe state of miners, and unsafe behavior of miners, which were the main risk factors affecting miners’ unsafe behavior in the deep coal mine. The improved model also divided the types of unsafe behaviors into five types: decision error, skill error, perception error, routine violation, and exceptional violation, as shown in Figure 2.

### 2.3. Structural Equation Model (SEM)

#### 2.3.1. System Variables and Hypothesis

SEM is a statistical method used to analyze the relationship between variables through a covariance matrix. As an empirical analysis model, it can test the internal structural relationships between the variables in the model. This relationship was expressed as a causality model and path graph through factor, path, and covariance analyses [44]. The SEM includes potential and observational variables. Observation variables can be obtained through direct observation, whereas potential variables need to be obtained indirectly through observation variables [45]. Based on the improved HFACS-CM model, the subsystems that affect the level of miners’ unsafe behavior are classified into four subsystems: environmental factors subsystem, organizational influence subsystem, unsafe supervision subsystem, and miners’ unsafe state subsystem. Therefore, the environmental factors subsystem, organizational influence subsystem, unsafe supervision subsystem, miners’ unsafe state subsystem, and miners’ unsafe behavior subsystem are considered latent variables in the structural equation model. Among them, the observed variables of the environmental factor subsystem include the physical environment, technical environment, and policy environment. The observed variables of the organizational influence subsystem include resource management, organizational climate, and organizational processes. The observed variables of the unsafe supervision subsystem include improper operation plan, failure to correct a problem, and violation supervision; the observed variables of the miners’ unsafe state subsystem include mental state, physiological state, and business ability. The observed variables of the miners’ unsafe behavior subsystem include decision errors, skill-based errors, perceptual errors, routine violations, and exceptional violations, as shown in Table 1.

Based on the HFACS-CM model, this study established a hypothetical relationship between the unsafe behavior of deep coal miners and various risk factors as follows:

**H1.**  *Environmental factors have a positive impact on the unsafe behavior of miners.*

**H2.**  *Organizational influence has a positive impact on the unsafe behavior of miners.*

**H3.**  *Unsafe supervision has a positive impact on the unsafe behavior of miners.*

**H4.**  *The unsafe state of miners has a positive impact on the unsafe behavior of miners.*

**H5.**  *Environmental factors have a positive impact on organizational influence.*

**H6.**  *Environmental factors have a positive impact on unsafe supervision.*

**H7.**  *Environmental factors have a positive impact on the unsafe state of miners.*

**H8.**  *Organizational influence has a positive impact on unsafe supervision.*

**H9.**  *Organizational influence has a positive impact on the unsafe state of miners.*

**H10.**  *Unsafe supervision has a positive impact on the unsafe state of miners.*

**H11.**  *Environmental factors positively affect the unsafe behavior of miners through the mediating effect of the unsafe state of miners.*

**H12.**  *Organizational influence positively affects the unsafe behavior of miners through the mediating effect of the unsafe state of miners.*

**H13.**  *Unsafe supervision positively affects the unsafe behavior of miners through the mediating effect of the unsafe state of miners.*

**H14.**  *Environmental factors positively affect the unsafe behavior of miners through the mediating effect of organizational influence.*

**H15.**  *Environmental factors positively affect the unsafe behavior of miners through the mediating effect of unsafe supervision.*

**Figure 3:**
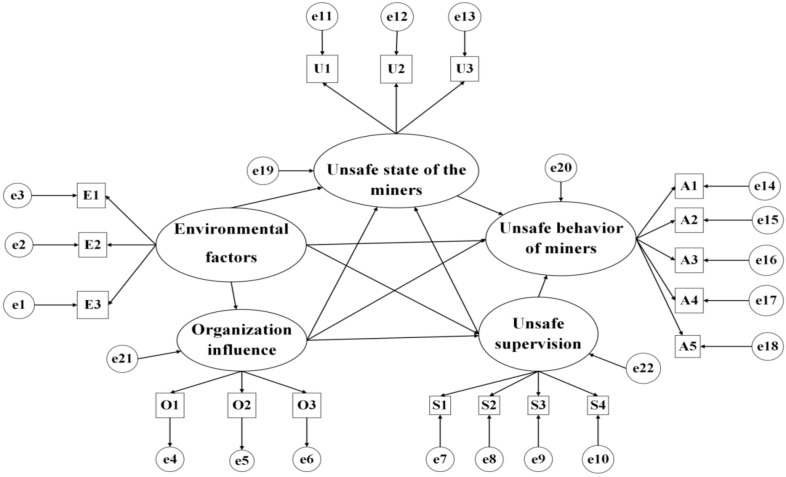
Preliminary SEM of the formation mechanism of unsafe behavior of deep coal miners.

According to the above assumptions, the initial structural model of the formation mechanism of the unsafe behavior of deep coal miners is shown in Figure 3.

#### 2.3.2. Questionnaire and Data Validation

(1) Questionnaire

Based on the above definitions of latent and observed variables, this study created a questionnaire that included each observed variable, and the questionnaire scale is shown in Table 2. Since the questionnaire ranked the importance of 18 observation variables in the SEM, a 5-point Likert scale was used to measure the significance of different observation variables under the same latent variable. From low to high, questionnaire respondents used scores of 1, 2, 3, 4, and 5, with 5 points representing very important, 4 points as relatively important, 3 points as general, 2 points as relatively unimportant, and 1 point as very insignificant. Before the questionnaire was distributed, according to the principles of convenience and economy, this study contacted three coal mines that had already carried out deep mining. The mining depth was greater than 600 m in all three mines. After communicating with the relevant persons in charge, 1150 questionnaires were sent to the three coal mines between September 2021 and February 2022 in electronic and paper forms. Paper questionnaires were used for offline surveys, and electric questionnaires were used for online surveys. The questionnaire was distributed to front-line managers in charge of deep mining and frontline workers who participated in deep mining. At the end of the survey, the research group collected 981 completed questionnaires, with a recovery rate of 85.3%. Nine hundred thirty-one valid samples meeting the survey requirements were obtained, with an effective rate of 80.96%. Table 3 shows the survey results on the sociodemographic variables of the effective samples, and Table 4 shows the statistical results of the survey.

(2) Reliability and Validity Verification

Reliability refers to the consistency of the answers of different respondents in the same questionnaire. Cronbach’s α test is widely used to evaluate the reliability of scale items [58]. Cronbach’s α coefficient is between 0 and 1. The higher the coefficient, the more reliable the questionnaire. A Cronbach’s α coefficient greater than or equal to 0.7 indicates good internal consistency of the scale. The closer the Cronbach’s α coefficient is to 1, the higher the reliability level of the scale. Cronbach’s α should not be lower than 0.5 [59]. Each variable in the survey scale must be measured using multiple items in multiple dimensions, and the reliability of each measurement must be tested. Cronbach’s α coefficient was adopted in this study to test the internal consistency of the items in the scale. During the test, Cronbach’s α coefficient significantly increased when the correlation coefficient between the revised item and the overall population was lower than 0.3 or when an item was deleted, indicating that this item should be considered for removal. Therefore, Cronbach’s α coefficient was used in this study to test the consistency of latent variables in the questionnaire, and its calculation formula is as follows:

(1) $$\alpha \;=\;\frac{k}{k\;-\;1}\;\times \;(1\;-\;\frac{\sum_{i=1}^{k}Z_{i}^{2}}{Z_{T}^{2}})$$

In Equation (1), *k* is the total number of questions in the questionnaire, $Z_{i}^{2}$ is the variance within the score range of question *i*, and $Z_{i}^{2}$ is the variance of the total score of all questions.

Validity refers to the correctness and quality of the questionnaire data, and whether the measurement method adopted in this study can accurately measure the content required in the scale. Content and structural validity are often used to test the overall validity of a survey scale. Most of the items in the survey scale of unsafe behaviors and risk factors for deep coal miners developed in this study were adapted from a mature scale developed by domestic and foreign scholars [2,8,10,14,42,46,47,48,49,50,51,52,53,54,55,56,57]. The self-compiled items were also modified several times based on literature analysis and expert consultation, combined with the actual production situation of deep coal mines. Therefore, the scale constructed in this study has certain content validity. The Kaiser-Meyer-Olkin (*KMO*) test, Bartlett sphericity test, and exploratory factor analysis are usually used to test a scale’s structural validity. When the *KMO* value is close to 1, and the Bartlett sphericity test significance level is less than 0.05, the data are suitable for factor analysis. The formula used to calculate the *KMO* value is as follows:

(2) $$KMO\;=\;\frac{\sum \sum_{i\neq j}r_{ij}^{2}}{\sum \sum_{i\neq j}r_{ij}^{2}\;+\;\sum \sum_{i\neq j}p_{ij}^{2}}$$

In Equation (2), $r_{ij}$ is the correlation coefficient between variables i and variable j, and $p_{ij}$ is the partial correlation coefficient between variables i and j.

SPSS 23 software was used to test the reliability and validity of the measurement items in the SEM, and the analysis results are shown in Table 5. It can be seen from the table that the reliability and validity of this scale are acceptable, and factor analysis can be carried out.

#### 2.3.3. SEM Fit Evaluation

**Figure 4:**
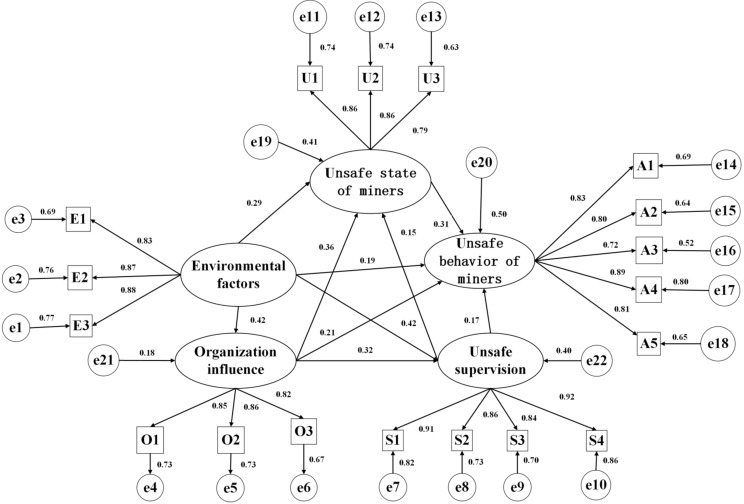
Standardized SEM of the formation mechanism of deep coal miners’ unsafe behavior.

The fitting test of the model determines whether the fitting effect of the data on the constructed model meets the requirements through certain fitting indices. The judgment criteria for each fit index are presented in Table 6. CFI, TLI, NFI, IFI, and GFI must be greater than or equal to 0.9, and the closer they are to 1, the better. The value of x^2^/df ranged from one to five. Root Mean Square Error of Approximation (RMSEA) should be less than or equal to 0.08 [60,61]. Amos 23 was used to test the goodness of fit of the hypothesis model. The calculation results for each indicator are listed in the third column of Table 6. As shown in Table 6, each fitting index of the initial hypothesis model constructed in this study was within the ideal range, indicating that the research data obtained in this study had an excellent fit effect on the model. After the original survey data were imported into Amos 23, the maximum likelihood estimation method was used to solve the SEM, and standardized path coefficients were obtained, as shown in Figure 4.

#### 2.3.4. Mediating Effect Test

In this study, the Bootstrap Algorithm in AMOS 23 was used to test the mediating effect of the unsafe state, organizational influence, and unsafe supervision of miners. The samples were repeated 5000 times, and 95% confidence intervals were calculated. The results of the data calculations are listed in Table 7. Table 7 shows that miners’ unsafe state has a mediating effect on environmental factors, organizational influence, unsafe supervision, and miners’ unsafe behavior. Both organizational influence and unsafe supervision mediate environmental factors and miners’ unsafe behaviors. A comparison of the standardized direct and indirect influence coefficients in Table 7 shows that the direct influence of environmental factors, organizational influence, unsafe supervision, and the unsafe state of miners on unsafe mining behaviors is more significant than their indirect influence. Environmental factors, organizational influence, unsafe supervision, and the unsafe state of miners have a much more direct impact on the unsafe behavior of miners than indirect influence, and the immediate effect plays a significant role.

#### 2.3.5. Standardized Regression Coefficients

Given the above analysis, this study only conducted a simulation analysis on the direct influence of environmental factors, organizational influence, unsafe supervision, and the unsafe state of miners on miners’ unsafe behavior. The obtained normalized path coefficients of SEM were converted into normalized weights of latent and observed variables, as shown in Table 8. This can be seen from Table 8 that the unsafe state of miners has the most significant weight, followed by organizational influence, environmental factors, and unsafe supervision.

### 2.4. System Dynamics Model (SD)

#### 2.4.1. Develop Causal Cycle Diagrams

**Figure 5:**
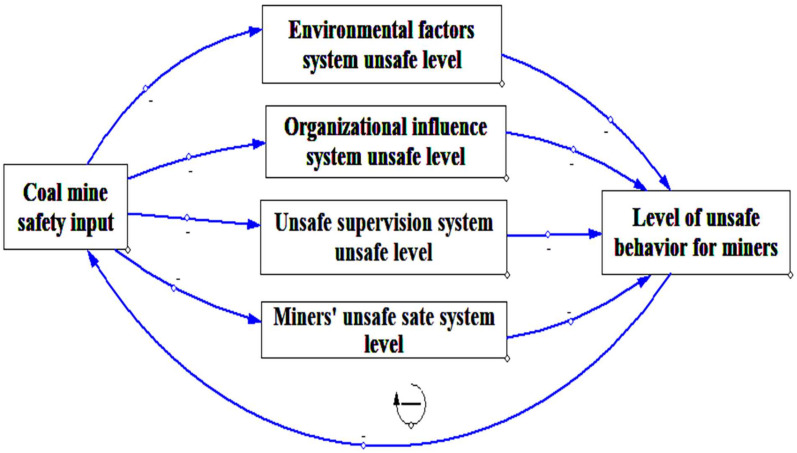
Causal cycle diagram of management of deep coal miner’s unsafe behaviors.

A causal cycle diagram is composed of one or more feedback cycles, which reflect the relationship between the inputs and outputs of various factors in the system, and the relationship between the external environment and the inputs and outputs of the system [64]. The coal mine safety input is an important guarantee for reducing the unsafe behavior level of deep coal miners. If miners’ unsafe behaviors are high, input in coal mine safety should be increased to improve the factors affecting miners’ unsafe behaviors. In contrast, if the level of unsafe behavior of miners is low, input in safety can be appropriately slowed down. From the perspective of the input and output of coal mine safety, this study analyzes the internal causal relationship and evolution process among the risk factors of unsafe behavior in deep coal miners. Based on the SEM constructed above, the deep coal mine safety input was introduced to establish the causal cycle of the safety management process, reflecting the unsafe behaviors of deep coal miners, as shown in Figure 5. As seen in Figure 5, increasing the input in coal mine safety can reduce the unsafe level of the environmental factors, organizational influence, unsafe supervision, and miner’s unsafe state subsystems, thus reducing the level of miners’ unsafe behavior. When the unsafe behavior of miners reaches the target level, the system can reduce the input of coal mine safety and avoid wasting resources through feedback on the level of unsafe behavior. In this system, coal mine safety input refers to the measures taken by coal mine enterprises to reduce unsafe behaviors. The system’s output relates to a reduction in the occurrence of unsafe behaviors.

#### 2.4.2. Formation of the SD Diagram and Model Check

**Figure 6:**
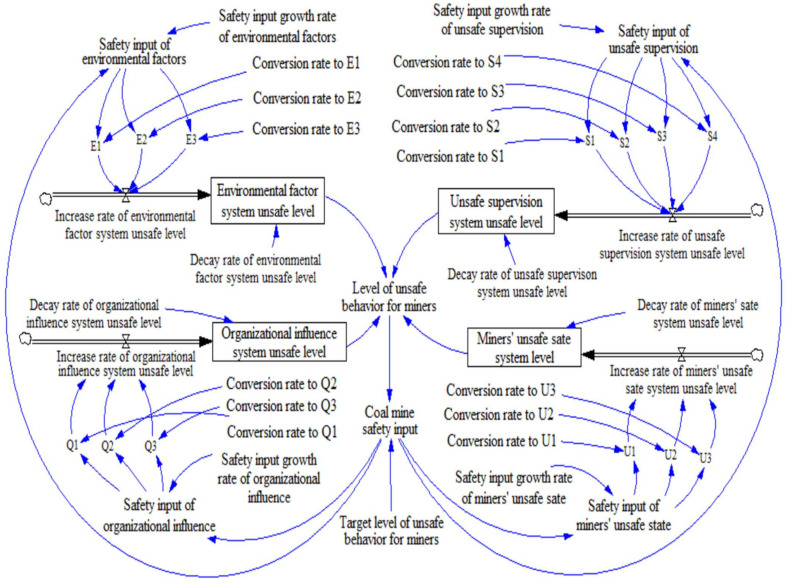
Inventory flow chart for the management of unsafe mining behaviors.

This study built a stock-flow chart based on the causal cycle chart. Every causal loop in an SD model should have at least one stock. Otherwise, there will be no accumulation. Stock refers to the value or level accumulated by a dynamic system’s core variables, reflecting the system’s changing state. According to the dynamic safety management system of deep coal miners’ unsafe behavior, the system stock is the unsafe behavior level of deep coal miners. Flow refers to the activity or flow that changes the inventory, and only flow can change the inventory value because all variables in the SD model change over time. According to the causality diagram and defined variables, the stock-flow diagram of the SD model of unsafe behavior level of deep coal miners was obtained, as shown in Figure 6, which includes 4 level variables, 4 rate variables, 18 auxiliary variables, and 21 constants. In Figure 6, you can identify the variables and corresponding equations that describe the system’s structure and control the relationship between the variables. Since the variables in the model were qualitative, they were measured in dimensionless units. The path coefficients and weights of the direct influence of various risk factors on the unsafe behaviors of deep coal miners in the SEM analyzed above are shown in Table 8. The SD model constructed in this paper takes the weight of each factor as the coefficient for calculation, and the mathematical expressions among variables in the SD model are shown in Table 9.

The formulas EFUL0, OIUL0, USUL0, and MUSL0 represented the initial values of the unsafe level of environmental factors, organizational influence, unsafe supervision, and the miner’s unsafe state, respectively. After determining the equation, the next step was to test the SD model, including a running test and a unit test, to verify the rationality of causality, the equation’s accuracy, and the unit’s consistency. After testing, the SD model worked well.

#### 2.4.3. Model Test

**Figure 7:**
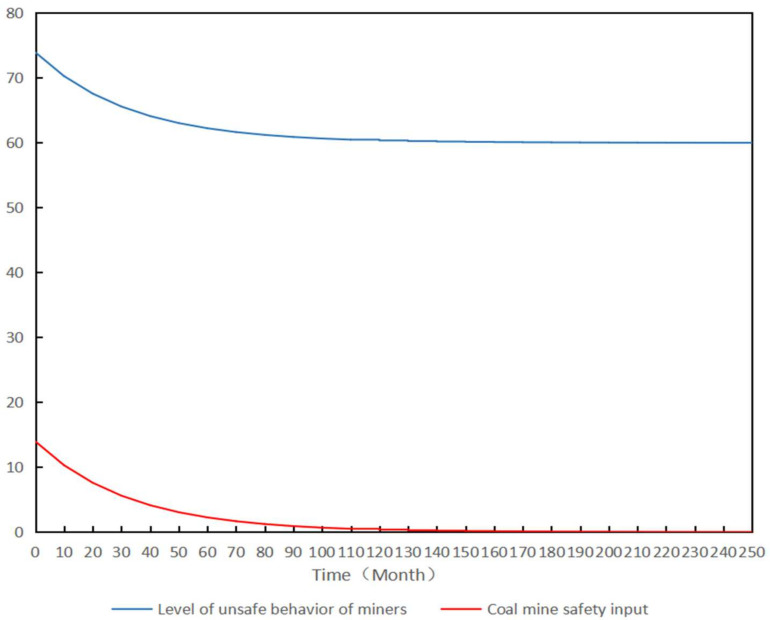
Dynamic influence of coal mine safety input on the level of miners’ unsafe behavior.

This study tested the SD model developed using Vensim Personal Learning Edition (PLE) software. The parameters of the established SD model can be divided into two categories: the initial value of the horizontal variable and the constant used for sensitivity adjustment. The initial values of the SD model were determined using expert scoring. In terms of the initial value of the level variable, according to the total score of 100, the unsafe behavior level of the deep coal miners is classified as excellent (≤60), good (60–69), medium (70–79), Pass (80–89), poor (≥90), as the scoring criteria. The respondents included 15 professionals, including professors and associate professors in the research field of coal mine safety management, deep coal mine safety managers, and deep mine front-line safety personnel. Therefore, the initial value of the unsafe level of environmental factors, organizational influence, unsafe supervision, and miners’ unsafe state was determined by experts as 80, 85, 85, and 80, respectively. For the constants in the SD model, the decay rate was set to 0.001, the safety input growth rate was set to 0.3, and the conversion rate was set to 0.1 for sensitivity analysis. The equations and parameters above were substituted into the established SD model. The simulation time was set to 250 months, the simulation step was set to one month, and the target level of unsafe behavior of miners was set to 60. The simulation results were then obtained by running Vensim PLE software. Figure 7 shows the dynamic relationship between the unsafe behaviors of the deep coal miners and input in coal mine safety.

As shown in Figure 7, the initial value of the unsafe behavior level of deep coal miners was approximately 73.91 under the initial coal mine safety input. With the influence of coal mine safety input on environmental factors, organizational influence, unsafe supervision, and miners’ unsafe state, miners’ unsafe behavior gradually decreased. At the unit time of the 190th, the miners’ unsafe behavior approached the target level of 60. If the level of unsafe behavior of miners does not reach the target level, the SD model adjusts the coal mine safety input according to the deviation between the target and actual levels. With a decrease in miners’ unsafe behavior, the corresponding input in coal mine safety will gradually decrease. When the level of miners’ unsafe behavior reaches the target level, the coal mine safety input is not zero because of the time lag between the safety input of coal mines and the resulting level of unsafe behavior of deep coal miners.

## 3. Results

### 3.1. Scenario Simulation and Sensitivity Analysis

**Figure 8:**
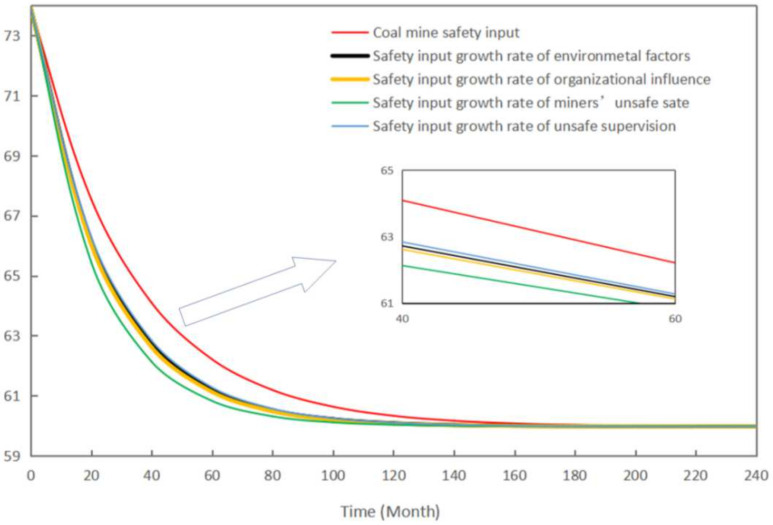
Dynamic influence of the increase rate of each subsystem safety input on the level of miners’ unsafe behavior.

This section uses the established SD model to simulate the contribution of each subsystem and the corresponding risk factors to the level of unsafe behavior of deep coal miners. In this study, each subsystem’s growth rate of safety input is set as shown in Table 10. The change in the growth rate of the safety input of each subsystem and the level of unsafe behavior of miners are shown in Figure 8.

As seen in Figure 8, increasing the growth rate of any subsystem’s coal mine safety input can reduce the level of deep coal miners’ unsafe behavior. However, the response rate of the unsafe behavior levels of miners varies. Among the subsystems, the safety input growth rate for miners’ unsafe state was the fastest in reducing miners’ unsafe behavior, followed by organizational influence, environmental factors, and unsafe supervision. The contribution rate (CR) was defined in this study to measure the sensitivity of each risk factor to the level of unsafe behavior of miners. The CR value is calculated as follows [65]:

(3) $$CR(A_{i})\;=\;\frac{\bar{CUBL(X_{i})}\;-\;\bar{CUBL(X_{0})}}{\bar{CUBL(X_{0})}}$$

In Formula (3), $\bar{CUBL\left(X_{0}\right)}$ is the average unsafe behavior level of miners affected before changing the influence factor X, and $\bar{CUBL\left(X_{i}\right)}$ is the average unsafe behavior level of miners after changing the impact factor X.

**Figure 9:**
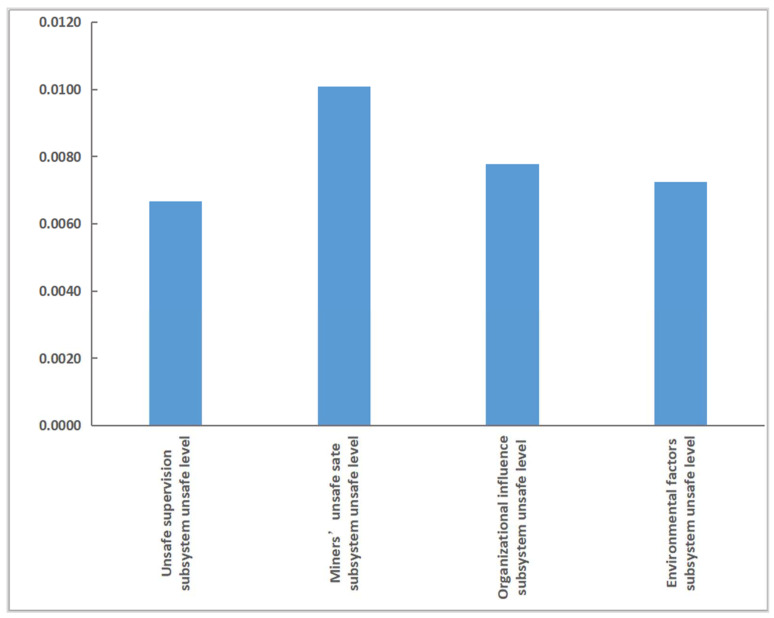
The CR value of the influence of each subsystem on the unsafe behavior of miners.

As shown in Formula (3), CR refers to the percentage of the average decrease in the unsafe behavior level of deep coal miners when the safety input conversion rate of a particular factor increases by a specific value, while other factors remain unchanged. Compared with the weight of each factor, the CR value can quantitatively express the contribution of the change in an element to the level of unsafe behavior of miners, which can provide a better basis for the decision-making of safety input in deep coal mines. According to the simulation results, the CR values of each subsystem of risk factors affecting the unsafe behavior of deep coal miners can be calculated using Equation (3), as shown in Figure 9. As seen in Figure 9, the CR value of increasing the growth rate of safety investment of each subsystem on the level of unsafe behavior of miners is in the following order: unsafe state of miners > organizational influence > environmental factors > unsafe supervision. Under the same growth rate of safety input, the CR value of the unsafe state to the unsafe behavior level of miners is higher than that of the other three subsystems, which indicates that the unsafe state of miners is the critical subsystem affecting the level of unsafe behavior of miners.

**Figure 10:**
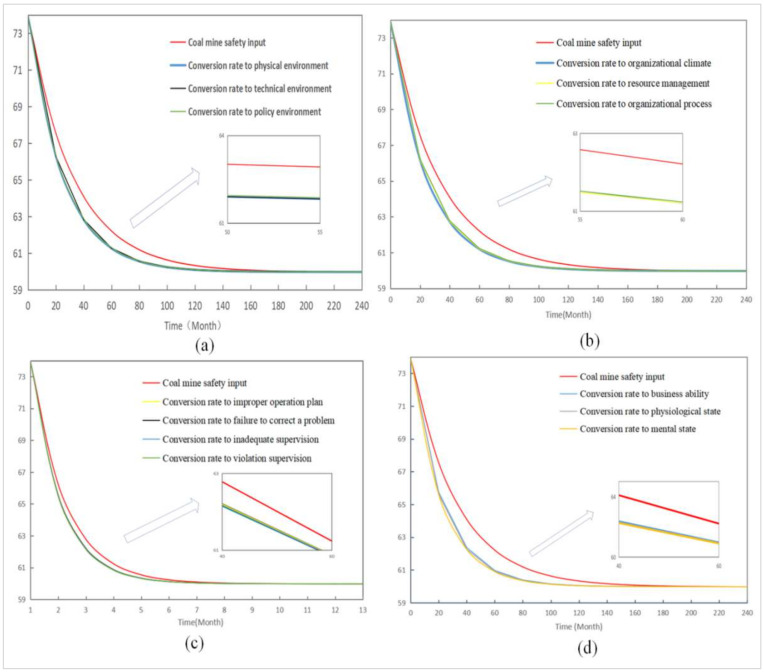
Levels of miners’ unsafe behavior with different conversion rates of risk factors: (**a**) Environmental factors; (**b**) Organizational influence; (**c**) Unsafe supervision; (**d**) Miners’ unsafe state.

The different conversion rates of risk factors in each subsystem are related to the control measures for the unsafe behaviors of coal miners. To further analyze the contribution of various control measures to reducing the level of unsafe behavior of miners, the conversion rate of each risk factor was increased to 0.5. At the same time, the other variables remained unchanged (the initial value was 0.1). The corresponding response level of the unsafe behavior of miners is shown in Figure 10. Figure 10 also shows that increasing the conversion rate of all risk factors can reduce the level of unsafe behavior of miners. For example, in the unsafe state subsystem of miners, increasing the conversion rate of the “mental state” can reduce the level of unsafe behavior of miners in the shortest time.

**Figure 11:**
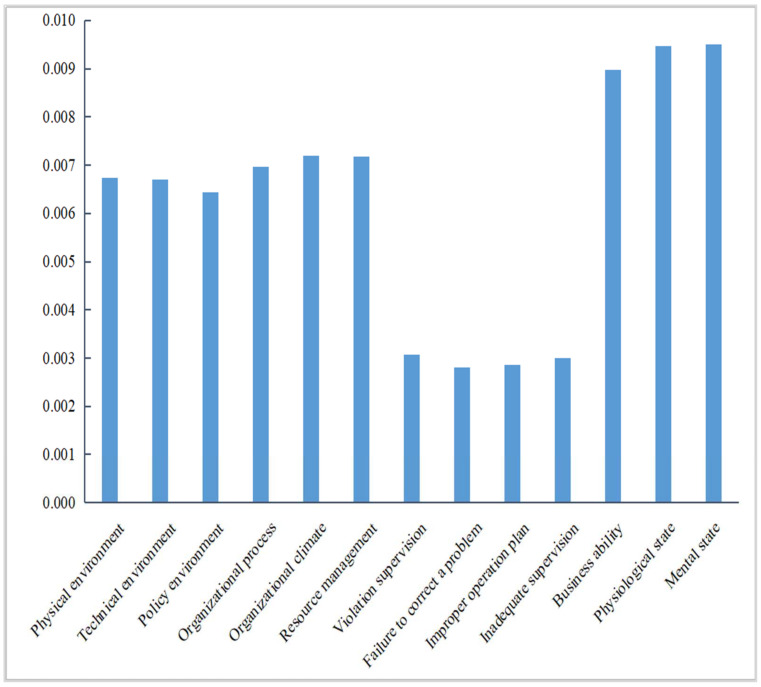
The CR value of the influence of risk factors on miners’ unsafe behavior.

The CR value of each risk factor for the level of unsafe behavior of miners is shown in Figure 11. As seen in Figure 11, mental state, physiological state, business ability, safety atmosphere, and resource management are the top five risk factors affecting the level of unsafe behavior of deep coal miners. Improving the conversion rate of these risk factors can rapidly reduce the unsafe behavior of deep coal miners, so coal mine enterprises should increase the safety input of the miner’s unsafe state subsystem. It is also necessary to improve the safety input of mental state, physiological state, business ability, safety atmosphere, resource management, and other key risk factors to rapidly reduce the level of unsafe behavior of the miners.

### 3.2. Intervention Strategies for the Unsafe Behavior of Deep Coal Miners

As seen from the above simulation results, adopting a combination intervention strategy to intervene in the unsafe behavior of deep coal miners can significantly reduce the unsafe behavior of coal miners. Therefore, this study proposes prevention and control measures for each risk factor, as shown in Table 11. These prevention and control measures can help managers of coal mine enterprises formulate a combination intervention strategy for the unsafe behavior of miners and reduce the unsafe behavior of miners quickly.

### 3.3. Weight of Risk Factors for the Unsafe Behavior of Deep Coal Miners

Based on the above simulation results and the CR value of each risk factor on the unsafe behavior level of deep coal mine workers, this study revised the weight of each risk factor, as shown in Table 12. As seen in Table 12, the top five most important factors affecting the unsafe behavior of deep coal miners are mental state, physiological state, business ability, organizational climate, and resource management. This finding is consistent with the results of this study, indicating that these factors are the most critical risk factors affecting the unsafe behavior of miners. Among the four subsystems of environmental factors, organizational influence, unsafe supervision, and the unsafe state of miners, the unsafe state of miners is the most important factor affecting miners’ unsafe behavior. In the subsystem of environmental factors, the physical environment was the most critical risk factor. The organizational climate was the most critical risk factor in the organizational influence subsystem. In the subsystem of unsafe supervision, violation supervision was the most critical risk factor. A miner’s mental state was the most critical risk factor in a miner’s unsafe state subsystem.

## 4. Discussion

This study developed a comprehensive evaluation method combining the HFACS-CM, SEM, and SD models to evaluate the dynamic relationship between miners’ unsafe behavior in deep coal mines and risk factors. Although there are many studies on the risk assessment of unsafe behaviors of coal miners, there are few studies on reducing the level of unsafe behaviors of deep coal miners from the perspective of the input and output of coal mine safety. Previous studies have shown that miners’ unsafe behavior is the main factor causing coal mine accidents. By analyzing the influencing factors of miners’ unsafe behavior and evaluating the risk level of miners’ unsafe behavior, scholars get the control measures of coal mine enterprises to miners’ unsafe behavior [66,67]. Compared with previous research on the unsafe behavior of coal miners, this study has the following advantages.

(1) This study enriches the content of coal mine safety management and fills the research gap regarding the “unsafe behavior of miners” in deep coal mine safety management. Previous studies have often discussed the unsafe behavior of shallow coal miners [68]. Compared to shallow mines, deep mines’ inner and outer environments have changed dramatically. The dynamic evaluation of the unsafe behaviors of miners in such environments can fill the research gap regarding the unsafe behaviors of miners in the safety management of deep coal mines and provide theoretical guidance for the management of the unsafe behaviors of deep coal miners.
(2) The combination of static and dynamic evaluations enriched the research methods in the field of coal mine workers’ unsafe behavior. Previous studies evaluating coal mine workers’ unsafe behavior have often adopted a single static or dynamic assessment [28]. This single static or dynamic evaluation method can only unilaterally evaluate the unsafe behavior of miners. Because individual miners have subjective initiative, their choice of unsafe behavior results from the interaction between themselves and the external environment. However, both the conditions of miners themselves and the internal and external mine environments will change over time, affecting miners’ unsafe behavior. In this study, the HFACS-CM model and SEM were used to analyze the interaction between the risk factors of the unsafe behavior of deep coal miners from a static perspective. The SD model was used to analyze the sensitivity of risk factors to the unsafe behavior of deep coal miners from a dynamic perspective. The combination of static and dynamic evaluation makes the research results of this study more practical and reasonable.
(3) Qualitative and quantitative methods are used to analyze the interaction between miners’ unsafe behavior and risk factors, making the simulation results more reliable. In previous studies, scholars used various methods to explore the relationship between miners’ unsafe behavior and risk factors and conducted dynamic simulation analyses based on the SD model [9]. However, the relationship between the elements in the previous simulation system was established based on certain assumptions, making the simulation results lacking a scientific nature. The relationship between the elements in the simulation system in this study was based on the HFACS-CM model and SEM analysis results, which makes establishing the simulation system more scientific and the obtained simulation results more reliable. The new method will enrich the theoretical approach to coal mine safety evaluation and promote a comprehensive assessment of the unsafe behaviors of deep coal miners, from theory to a broad application level.

## 5. Conclusions

Based on the HFACS-CM, SEM, and SD models, this study proposed a comprehensive evaluation model for deep coal miners’ unsafe behavior that analyzed the static and dynamic effects of each risk factor on the unsafe behavior of coal miners. The main conclusions are as follows.

(1) Environmental factors, organizational influence, unsafe supervision, and the unsafe state of miners were the main risk factors affecting the unsafe behavior among deep coal miners. These risk factors had a significant direct and indirect influence on the unsafe behavior of miners, and their direct impact was far more critical than their indirect influence. The unsafe state of miners had mediating effects on environmental factors, organizational influence, unsafe supervision, and miners’ unsafe behavior. Organizational influence and unsafe supervision also mediated environmental factors and miners’ unsafe behavior. These results show that environmental factors, organizational influence, unsafe supervision, and the unsafe state of miners have different degrees of impact on the unsafe behavior of miners in deep coal mines. The formation of unsafe behavior is the result of their interaction. These research results also further demonstrate that the relevant hypotheses are valid. In the deep coal mine, due to the deepening of mining depth, the temperature, humidity, ground stress, noise, dust, and other environmental factors in the mine become more severe. Working in such a harsh environment for a long time will seriously affect the safety state of miners’, such as physiology, psychology, and business skills. Fatigue, mental tension, inattention, and other unsafe conditions easily cause miners to exhibit various unsafe behaviors, which increases risks in the deep mining process. The severe environmental factors in deep coal mines will also have a particular impact on the organization, management, and safety supervision of coal mining enterprises and will then affect the safety state of miners, leading to the formation of unsafe behaviors. The main reason is that the environmental factors in deep coal mines are complex and changeable, and the previous prevention and control measures of miners’ unsafe behaviors are no longer applicable in this environment. For example, the training content is no longer appropriate for deep mines. In addition, in deep mining, the lack of supervision of miners’ behavior leads to fluke, shirking responsibility, carelessness, and other unsafe states, leading to miners’ unsafe behavior. Therefore, prevention and control measures, such as improving the environmental factors in deep coal mines, strengthening the organization’s safety management, and strengthening the supervision of miners’ behavior, can effectively improve the miners’ unsafe state and reduce the risk of miners’ unsafe behavior.
(2) The unsafe state of miners in the deep coal mine has a significant positive impact on their unsafe behavior, which is also the most important risk factor affecting their unsafe behavior. Various prevention and control strategies for miners’ unsafe states can quickly reduce the level of miners’ unsafe behavior and reduce the risk of unsafe behavior. This research result is consistent with the research conclusions of previous scholars [23,69]. A long-term working environment of the deep coal mine and complex production process will cause negative changes in miners’ safe state. Their unsafe states, such as anxiety, depression, fatigue, and lack of safety knowledge, lead to unsafe behaviors. Therefore, taking appropriate prevention and control measures to improve the unsafe state of miners can more directly reduce the risk of unsafe behavior of miners.
(3) The physical environment was the most critical risk factor in the subsystem of environmental factors. The organizational climate was the most critical risk factor in the subsystem of organizational influence, inadequate supervision was the most critical risk factor in unsafe supervision, and mental state was the most critical risk factor in the unsafe state of miners. The optimal coal mine safety input aimed at these risk factors can quickly reduce the level of unsafe behavior of miners. Deep coal mines’ environmental factors mainly include physical, technical, and policy environments. Among them, physical environmental factors refer to high temperature, high humidity, high ground stress, noise, etc. Taking steps to improve these factors can quickly reduce the risk of miners’ unsafe behavior. “Organization influence” mainly includes organizational processes, climate, and resource management. Organizational climate is the most critical organizational factor affecting miners’ unsafe behavior. Strengthening the construction of a safety culture in coal mine enterprises and creating an excellent organizational climate can reduce the risk of miners’ unsafe behavior. Unsafe supervision mainly includes violation supervision, improper operation plans, failure to correct a problem, and inadequate supervision. Inadequate supervision mainly refers to managers’ lack of guidance for the miners’ work and their failure to intervene in the miners’ unsafe behaviors. Therefore, strengthening the guidance of miners’ work and increasing the intervention of miners’ unsafe behavior can quickly reduce the risk of miners’ unsafe behavior. The unsafe state of miners in the deep coal mine mainly refers to their mental state, physiological state, and business ability. Mental state refers mainly to nervousness, anxiety, fluke psychology, etc. According to the research results, taking corresponding measures to improve the adverse mental states of miners can quickly reduce the risk of miners’ unsafe behavior.
(4) Mental state, physiological state, business ability, organizational climate, and resource management were the top five risk factors affecting the level of unsafe behavior among deep coal miners. Prevention and control measures against these risk factors can reduce the risk of miners’ unsafe behavior in deep coal in the shortest time. Therefore, taking measures to improve the miners’ unhealthy physical and psychological state, improve the miners’ business abilities, create an excellent organizational safety atmosphere, and strengthen the management of resources can quickly reduce the risk of miners’ unsafe behavior and reduce the occurrence of coal mine accidents.
(5) Compared with the single intervention strategy, the combined intervention strategy that improves environmental factors, organizational influence, unsafe supervision, and the unsafe state of miners can reduce the risk of miners’ unsafe behavior more quickly. Therefore, in formulating prevention and control measures for miners’ unsafe behaviors in deep coal mines, coal mining enterprises need to consider environmental factors, supervision, management factors, and miners’ safety status factors to rapidly reduce the risk of unsafe behaviors among miners.

## 6. Suggestions

According to the analysis results of HFACS-CM and SEM, environmental factors, organizational influence, unsafe supervision, and the unsafe state of miners are the main risk factors leading to miners’ unsafe behavior. The formation of miners’ unsafe behavior results from the interaction between these risk factors. According to the simulation results of the SD model, adopting an appropriate unsafe behavior intervention strategy can reduce the risk of miners’ unsafe behavior. To sum up, according to the research conclusions of the HFACS-CM-SEM-SD model and combined with the actual situation of miners’ unsafe behaviors in the deep coal mine, this study constructed a prevention and control system of miners’ unsafe behaviors in four aspects: environmental factors, organizational influence, unsafe supervision, and unsafe state of miners, and formulated specific prevention and control measures of unsafe behaviors.

(1) Environmental factors

The environmental factors that affect deep coal miners’ unsafe behavior mainly include physical, technical, and policy environments. Therefore, this paper takes measures to intervene in the unsafe behavior of miners from the following aspects.

Improve the physical environment of the deep coal mine and create a good working atmosphere. Extreme physical environment factors, such as high temperature, high pressure, and high humidity in deep coal mines, will seriously affect miners’ physical and mental states and lead to unsafe behaviors. Managers can take the following measures to improve the physical environmental factors in the deep coal mine. ① Introduce professional talents to carry out scientific coal seam geological exploration. ② Use advanced cooling technology to improve the climate environment in the deep coal mine. ③ Muffling measures are adopted to reduce downhole noise and strengthen the safety management of downhole noise. ④ Maintain good lighting intensity in the deep coal mine and equipped with emergency lighting equipment.

With the development of information technology, an excellent technical environment is also an important guarantee to reduce the risk of unsafe behavior of deep coal miners. Managers can take measures from the following aspects to improve the technical environment of deep coal mines. ① Introduce advanced coal mining technology and build intelligent mines. ② Introduce advanced protective equipment and improve the supervision and management system for safety protective equipment. ③ Introduce advanced machinery and equipment, and improve the machinery and equipment management system. ④ Introduce intelligent technology and methods to establish a warning and prevention system for underground environments in deep coal mines.

A good policy environment can also reduce the risk of miners’ unsafe behavior in deep coal mines. Managers can take the following measures to intervene in miners’ unsafe behavior and reduce the miners’ risk of unsafe behavior. ① Improve laws and regulations on deep coal mines’ safety and standardize deep coal mines’ safety management systems. ② Strengthen the rectification of small coal mines and end the deep mining of coal mines without a deep mining capacity. ③ Increase incentives for coal mining enterprises, give incentives to those mines with few accidents, and abide by laws, such as tax reduction and exemption. ④ Government supervision departments should improve the management system of deep mining of coal mining enterprises to prevent the occurrence of unsafe behaviors of coal miners from the source. ⑤ Government regulatory departments strictly examine the application data of coal mining enterprises for deep mining and firmly reject qualification applications that do not meet the conditions for deep mining. ⑥ Government regulatory departments can reasonably supplement the regulatory force by increasing the enterprise staff, contract staff, temporary staff, etc., to alleviate the grassroots safety regulatory personnel shortage. ⑦ Innovate safety supervision models for the coal mine, promote market-oriented supervision mechanisms, and give full play to the independence and autonomy of third-party regulatory bodies in the supervision process. ⑧ Establish a safety supervision information system for coal mines to improve the efficiency of government supervision. ⑨ Promote the professional process of coal miners and cultivate an experienced team of coal mining workers.

(2) Organization influence

In a deep coal mine, reducing the adverse influence of miners’ organizations can also effectively reduce the risk of miners’ unsafe behavior. According to the conclusion above, this study takes measures from the following aspects to reduce the risk of miners’ unsafe behavior. ① Strengthen the construction of an organizational safety culture and form good corporate safety values. ② Strengthen communication within the organization and improve team cohesion. ③ Strengthen safety education and training to improve miners’ professional skills. ④ Increase investment in safety and improve the system for preventing and controlling unsafe behaviors. ⑤ Strengthen organization safety management and improve the organization’s safety management system. ⑥ Perfect organizational structure, carry on good organizational division of labor. ⑦ Improve the safety reward and punishment system to improve miners’ job satisfaction.

(3) Unsafe supervision

Strengthening the supervision of miners’ unsafe behavior in a deep coal mine can reduce the risk of miners’ unsafe behavior. Managers can take the following measures to reduce the risk of miners’ unsafe behavior. ① Strengthen safety inspection and supervision and improve miners’ safety supervision system. ② Improve the internal safety management laws and regulations of coal mining enterprises. ③ Make a reasonable work plan. ④ Strengthen the prevention and investigation of the risk sources of unsafe behavior of miners. ⑤ Strengthen the education, training, supervision, and management of front-line managers.

(4) Unsafe state of the miners

Miners’ unsafe state in the deep coal mine directly impacts their unsafe behaviors, and other risk factors can indirectly affect miners’ unsafe behaviors through their mediating effect. In addition, the unsafe state of miners is the most critical risk factor affecting their unsafe behavior. Given this, managers can take measures from the following aspects to improve the unsafe state of miners and quickly reduce the risk of unsafe behavior. ① Cultivate miners’ safety values and enhance miners’ safety awareness. Coal mine enterprises can regularly organize and carry out safety knowledge sharing, safety knowledge competition, safety slogans, and other activities to help miners form correct safety values and enhance their safety awareness. ② The reasonable distribution of work tasks reduces the working pressure on workers. Reasonable working hours and workloads can reduce miners’ work pressure and avoid the risk of unsafe behaviors. ③ Establish a physical and mental health evaluation mechanism for miners. A suitable evaluation mechanism of miners’ physical and mental health can help managers understand the miners’ physical and psychological health status comprehensively and in a timely manner to adjust the work of workers unsuitable for underground work. ④ Strengthen professional skills training and improve miners’ safety literacy. Coal mining enterprises can improve the safety literacy of miners by broadcasting safety education videos, posting safety slogans, and organizing expert lectures. ⑤ Strengthen emergency drill mechanisms and innovate safety education and training methods. Coal mine enterprises should improve their disaster emergency drill mechanisms and organize more front-line workers to participate in disaster emergency drill activities. In addition, various innovative training methods should be introduced to enhance safety education and training effectiveness.

Through the HFACS-CM-SEM-SD model constructed, this study comprehensively evaluates the unsafe behaviors of miners in deep coal mines from three aspects: the risk factors of unsafe behaviors, the formation mechanism of unsafe behaviors, and the intervention strategy for unsafe behaviors. Specific prevention and control measures for miners’ unsafe behaviors are also formulated from four aspects: environmental factors, organizational influence, unsafe supervision, and the unsafe state of miners. The results of this study provide essential reference values for the risk assessment and safety management of miners’ unsafe behavior in deep coal miners. This study has important theoretical and practical significance. On a theoretical level, this study enriches the content of deep coal mine safety management. In the past, most research on disaster prevention in deep coal mines has been discussed from coal and gas outbursts, roof accidents, floods, etc., while few studies have been conducted from the perspective of miners’ unsafe behavior. This study fills a gap in this field. The research results discussed in this study can also provide some reference value for future scholars in this field. In addition, the research method adopted in this study offers a new idea for scholars to study the unsafe behavior of miners, which overcomes the difficulties of obtaining risk factors and exploring the relationship between them in the past study. On a practical level, this study can help coal mining enterprises improve their safety management ability in the deep mining process. The research results of this study can enable the safety managers of coal mining enterprises to better understand the formation process of miners’ unsafe behaviors in deep coal mines and help them formulate more effective prevention and control measures to reduce the risk of unsafe behaviors.

## Figures and Tables

**Table 1:** Potential variables and observed variables in the initial SEM model.

Latent Variables	Observation Variable	The Index Code
Environmental factors	Physical environment	E1
	Technical environment	E2
	Policy environment	E3
Organization influence	Resource management	O1
	Organizational climate	O2
	Organizational process	O3
Unsafe supervision	Violation supervision	S1
	Improper operation plan	S2
	Failure to correct a problem	S3
	Inadequate supervision	S4
Unsafe state of the miners	Mental state	U1
	Physiological state	U2
	Business ability	U3
Unsafe behavior of miners	Decision errors	A1
	Skill-based errors	A2
	Perceptual errors	A3
	Routine violations	A4
	Exceptional violations	A5

**Table 2:** Survey Scale of Unsafe Behavior and Risk Factors of Miners in the Deep Coal Mine.

Variable Name	Measuring Project	Items	Source
Demographic variable	Age	Q1	Wang [12], Paul [46]
	Education background	Q2	Zhang [47]
	Length of Service	Q3	Wang [2], Wang [12]
	Income	Q4	Zhang [48]
	Position	Q5	Liu [8]
	Marital status	Q6	Huang [49]
Environmental factors	Physical environment	Q7	Guo [50]
	Technical environment	Q8	Liu [8]
	Policy environment	Q9	Liu [8]
Organization influence	Resource management	Q10	Liou [51]
	Organizational climate	Q11	Liou [51], Casey [52]
	Organizational process	Q12	Liu [8]
Unsafe supervision	Violation supervision	Q13	Zhang [48]
	Improper operation plan	Q14	Liu [8]
	Failure to correct a problem	Q15	Tong [10]
	Inadequate supervision	Q16	Zhang [53]
Unsafe state of the miners	Mental state	Q17	Yu [9], Wang [54]
	Physiological state	Q18	Fa [55], Duma [56]
	Business ability	Q19	Liu [8]
Unsafe behavior of miners	Decision errors	Q20	Shappell [57]
	Skill-based errors	Q21	Shappell [57]
	Perceptual errors	Q22	Shappell [57]
	Routine violations	Q23	Shappell [57]
	Exceptional violations	Q24	Shappell [57]

**Table 3:** Demographic statistical results of valid samples.

Name	Category	Frequency	Percentage
Length of service	1 year or less	44	4.73
	1–3 years (including 3 years)	527	56.61
	3–5 years	273	29.32
	5 years or above	87	9.34
Age	18–24	63	6.77
	25–34	99	10.63
	35–44	444	47.69
	45 and above	325	34.91
Education background	Junior high and below	180	19.33
	High school or vocational high school	485	52.09
	College	174	18.69
	Undergraduate course	58	6.23
	Master’s degree or above	34	3.65
Income	3000 yuan or less	29	3.11
	3000–5000 yuan (including 5000)	293	31.47
	5000–10,000 yuan	363	38.99
	10,000 yuan and above	246	26.42
Position	General staff	569	61.12
	Grass-roots manager	323	34.69
	Middle manager	27	2.90
	Senior manager	12	1.29
Marital status	unmarried	239	25.67
	married	692	74.33

**Table 4:** Survey Results.

Item	Score				
	5	4	3	2	1
E1	32	161	279	284	175
E2	27	144	293	296	171
E3	17	159	245	307	203
O1	18	122	241	365	185
O2	24	103	295	311	198
O3	27	110	279	329	186
S1	59	126	162	235	349
S2	48	109	180	285	309
S3	38	176	156	294	267
S4	50	126	162	208	385
U1	27	142	218	378	166
U2	36	119	283	348	145
U3	35	96	302	316	182
A1	57	45	96	229	504
A2	36	75	220	269	331
A3	33	62	246	287	303
A4	36	76	131	266	422
A5	30	107	203	267	324

**Table 5:** Reliability and validity test results.

	Cronbach’s α	KMO Value	Bartlett’s Test of Sphericity	
			Approximate Chi-Square	Sig.
Standard	>0.9 as excellent, 0.7–0.8 as acceptable range.	>0.9 as excellent, 0.6–0.8 as acceptable range.	NA	<0.05
Results	0.933	0.922	12,341.514	0.000

Note: NA = Not Available.

**Table 6:** Model fitting index evaluation criteria and model fitting results.

Fitting Index	Standard	Source	Model Fitting Results	
X^2^/df	1≤ X^2^/df ≤ 5	Seo [62], Al-Mekhlafifi [63]	2.895	Qualified
GFI	0.9 ≤ GFI ≤ 1, and the closer it gets to 1, the better	Seo [62], Al-Mekhlafifi [63]	0.981	Very good
AGFI	0.9 ≤ AGFI ≤ 1, and the closer it gets to 1, the better	Seo [62], Al-Mekhlafifi [63]	0.943	Good
NFI	0.9 ≤ NFI ≤ 1, and the closer it gets to 1, the better	Seo [62], Al-Mekhlafifi [63]	0.971	Good
IFI	0.9 ≤ IFI ≤ 1, and the closer it gets to 1, the better	Seo [62], Al-Mekhlafifi [63]	0.981	Good
TLI	0.9 ≤ TLI ≤ 1, and the closer it gets to 1, the better	Seo [62], Al-Mekhlafifi [63]	0.976	Good
CFI	0.9 ≤ CFI ≤ 1, and the closer it gets to 1, the better	Seo [62], Al-Mekhlafifi [63]	0.981	Good
RMSEA	RMSEA ≤ 0.08	Seo [62], Al-Mekhlafifi [63]	0.045	Good

**Table 7:** Mediating effect test results.

Path	Direct Effect	Indirect Effect	*p*	95% Confidence Interval	
				Lower	Upper
Environmental factors → Unsafe state of miners → Unsafe behavior of miners	0.193	0.089	***	0.461	0.305
Organizational influence → Unsafe state of miners → Unsafe behavior of miners	0.212	0.109	***	0.152	0.269
Unsafe supervision → Unsafe state of miners → Unsafe behavior of miners	0.173	0.045	***	0.017	0.064
Environmental factors → Unsafe supervision → Unsafe behavior of miners	0.193	0.074	***	0.461	0.305
Environmental factors → Organizational influence → Unsafe behavior of miners	0.193	0.089	***	0.461	0.305
Environmental factors → Organizational influence → Unsafe state of miners → Unsafe behavior of miners	0.193	0.046	***	0.461	0.305
Environmental factors → Organizational influence → Unsafe supervision → Unsafe state of miners → Unsafe behavior of miners	0.193	0.006	***	0.461	0.305
Environmental factors → Unsafe supervision → Unsafe state of miners → Unsafe behavior of miners	0.193	0.019	***	0.461	0.305

Note: *** p < 0.001.

**Table 8:** Standardized path coefficients and corresponding weights.

Path	Normalized Path Coefficient	Weight
Unsafe behavior of miners ← Environmental factors	0.193	0.218
Unsafe behavior of miners ← Organization influence	0.212	0.239
Unsafe behavior of miners ← Unsafe supervision	0.173	0.195
Unsafe behavior of miners ← Unsafe state of miners	0.308	0.348
E1 ← Environmental factors	0.878	0.34
E2 ← Environmental factors	0.873	0.338
E3 ← Environmental factors	0.829	0.321
O1 ← Organization influence	0.853	0.337
O2 ← Organization influence	0.857	0.339
O3 ← Organization influence	0.82	0.324
S1 ← Unsafe supervision	0.906	0.257
S2 ← Unsafe supervision	0.855	0.243
S3 ← Unsafe supervision	0.837	0.238
S4 ← Unsafe supervision	0.925	0.263
U1 ← Unsafe state of miners	0.863	0.343
U2 ← Unsafe state of miners	0.858	0.341
U3 ← Unsafe state of miners	0.793	0.315
A1 ← Unsafe behavior of miners	0.83	0.205
A2 ← Unsafe behavior of miners	0.797	0.197
A3 ← Unsafe behavior of miners	0.721	0.178
A4 ← Unsafe behavior of miners	0.893	0.221
A5 ← Unsafe behavior of miners	0.808	0.2

**Table 9:** Variables and functions in the SD model.

Variable	Type	Abbreviation	Function
Level of unsafe behavior of miners	Auxiliary	LUBM	LUBM = 0.218 × EFUL + 0.239 × OIUL + 0.195 × USUL + 0.348 × MSUL
Target of unsafe behavior for miners	Constant	TUBM	*NA*
Coal mine safety input	Auxiliary	CMSI	CMSI = LUBM − TUBM
Environmental factor system unsafe level	Level	EFUL	$EFUL\;=\;INTEG\;\left(IREF\;-\;DREF,\;EFUL0\right)$
Increase rate of environmental factor system unsafe level	Rate	IREF	IREF = −0.34 × E1 + 0.338 × E2 + 0.321 × E3
Decay rate of environmental factor system unsafe level	Constant	DREF	*NA*
Safety input growth rate of environmental factors	Constant	SIREF	*NA*
Safety input of environmental factors	Auxiliary	SIEF	SIEF = CMSI × SIREF
Conversion rate to Ei,i = 1, 2,3	Constant	CREi	*NA*
Ei, i = 1, 2, 3	Auxiliary	Ei	Ei = CREi × SIEF
Organizational influence system unsafe level	level	OIUL	$OIUL\;=\;INTEG\left(IROI\;-\;DROI,\;OIUL0\right)$
Increase rate of organizational influence system unsafe level	Rate	IROI	$IROI\;=\;-\left(0.337\;\times \;O1\;+\;0.339\;\times \;O2\;+\;0.324\;\times \;O3\right)$
Decay rate of organizational influence system unsafe level	Constant	DROI	*NA*
Safety input growth rate of organizational influence	Constant	SIROI	*NA*
Safety input of organizational influence	Auxiliary	SIOI	SIOI = CMSI × SIROI
Conversion rate to Oi, i = 1, 2, 3	Constant	CROi	*NA*
Oi, i = 1, 2, 3	Auxiliary	Oi	Oi = CROi × SIOI
Unsafe supervision system unsafe level	Level	USUL	$USUL\;=\;INTEG\left(IRUS\;-\;DRUS,\;USUL0\right)$
Increase rate of unsafe supervision system unsafe level	Rate	IRUS	$IRUS\;=\;-\left(0.257\;\times \;S1\;+\;0.243\;\times \;S2\;+\;0.238\;\times \;S3\;+\;0.263\;\times \;S4\right)$
Decay rate of unsafe supervision system unsafe level	Constant	DRUS	*NA*
Safety input growth rate of unsafe supervision	Constant	SIRUS	*NA*
Safety input of unsafe supervision	Auxiliary	SIUS	SIUS = CMSI × SIRUS
Conversion rate to Si, i = 1, 2, 3, 4	Constant	CRSi	*NA*
Si, i = 1, 2, 3, 4	Auxiliary	Si	Si = CRSi × SIUS
Miners’ unsafe sate system level	Level	MUSL	$MUSL\;=\;INTEG\left(IRMS\;-\;DRMS,\;MUSL0\right)$
Increase rate of miners’ unsafe sate system level	Rate	IRMS	$IRMS\;=\;-\left(0.343\;\times \;U1\;+\;0.341\;\times \;U2\;+\;0.315\;\times \;U3\right)$
Decay rate of miners’ unsafe sate system level	Constant	DRMS	*NA*
Safety input growth rate of miners’ unsafe sate	Constant	SIRMS	*NA*
Safety input of miners’ unsafe sate	Auxiliary	SIMS	SIMS = CMSI × SIRMS
Conversion rate to Ui, i = 1, 2, 3	Constant	CRUi	*NA*
Ui, i = 1, 2, 3	Auxiliary	Ui	U1i = CRUi × SIMS

Note: *NA* = Not Available.

**Table 10:** Coal mine safety input scenarios for different subsystems.

Indicators	Simulation Scene				
	Scenario 1	Scenario 2	Scenario 3	Scenario 4	Scenario 5
Safety input growth rate of environmental factors (SIREF)	0.3	0.6	0.3	0.3	0.3
Safety input growth rate of organizational influence (SIROI)	0.3	0.3	0.6	0.3	0.3
Safety input growth rate of unsafe supervision (SIRUS)	0.3	0.3	0.3	0.6	0.3
Safety input growth rate of miners’ unsafe sate (SIRMS)	0.3	0.3	0.3	0.3	0.6

**Table 11:** Intervention strategies for unsafe behavior of deep coal miners.

Risk Factors		Coping Strategies	Specific Measures
Environmental factors	Physical environment	Improve physical environment	Strengthening protection; Strengthening exploration of complex geology; Create a good working environment
	Technical environment	Strengthening technical management	Perfect mechanical equipment; Improve safety monitoring system; Improve the ventilation system; Innovation of safe coal mining technology
	Policy environment	Strengthen government oversight and management	Strengthen the supervision of government departments on deep mining of coal mining enterprises
Organizational influence	Resource management	Strengthening resource Management	Strengthening safety education and training; Carry out reasonable staffing; Increase investment in security
	Organizational climate	Strengthening resource Management	Assigning reasonable production targets; Eliminate superpower production; Strengthen communication among members of the organization; Strengthen teamwork among workers; Improve team cohesion; Improve safety culture.
	Organizational process	Strengthen the management process of the organization	Improve the rules and regulations of the organization; Timely technical guidance; Improve workers’ operating procedures
Unsafe supervision	Inadequate supervision	Strengthen supervision	Strengthening guidance on workers’ behavior; Timely intervention of workers’ unsafe behavior
	Improper operation plan	Make reasonable operation plan	Rational organization of labor production; End blindly organized production; Develop reasonable operation plan
	Failure to correct a problem	Correct mistakes in time	Strengthening risk management capacity; Strengthening capacity for risk identification and assessment; Strengthen the investigation and management of potential safety hazards
	Violation supervision	Strengthen supervision and management	Strengthening safety inspection and supervision; Formulate reasonable reward and punishment system; Strictly abide by laws and regulations; Improve coal mine safety laws and regulations; Perfect safety supervision system; Improve the safety management system
Unsafe state of the miners	Mental state	Improve mental state	Strengthening safety education and training; Enhance safety awareness; Reduce work pressure; Psychological counseling
	Physiological state	Improve physiological state	Reasonable arrangement of workload; Prioritize rest
	Business ability	Enhance business capabilities	Strengthen personal business skills training; Strengthen personal safety literacy

**Table 12:** Index weights of risk factors for unsafe behaviors of deep coal miners.

First-Level Indicators	Weight of First-Level Indicators	Second-Level Indicators	CR Value	Weight of Second-Level Indicators
Environmental factors	0.2455	Physical environment	0.00673	0.0832
		Technical environment	0.00670	0.0828
		Policy environment	0.00644	0.0795
Organizational influence	0.2636	Organizational process	0.00696	0.0860
		Organizational climate	0.00720	0.0890
		Resource management	0.00717	0.0886
Unsafe supervision	0.1452	Violation supervision	0.00307	0.0379
		Failure to correct a problem	0.00281	0.0347
		Improper operation plan	0.00286	0.0354
		Inadequate supervision	0.00301	0.0372
Unsafe state of the miners	0.3456	Business ability	0.00897	0.1109
		Physiological state	0.00948	0.1171
		Mental state	0.00951	0.1176

## Data Availability

Requests for datasets can be sent to Xue Wang, xuewang2022@163.com.
